# Characterization of Induced Pluripotent Stem Cell Microvesicle Genesis, Morphology and Pluripotent Content

**DOI:** 10.1038/srep19743

**Published:** 2016-01-22

**Authors:** Jing Zhou, Shima Ghoroghi, Alberto Benito-Martin, Hao Wu, Uchenna John Unachukwu, Linda Saxe Einbond, Sara Guariglia, Hector Peinado, Stephen Redenti

**Affiliations:** 1 Department of Biological Sciences, City University of New York, Lehman College, 250 Bedford Park Boulevard West, Bronx, NY 10468; 2 Biology Program, The Graduate School and University Center, City University of New York, 365 5th Avenue, New York, NY 10016; 3 Departments of Pediatrics, Hematology/Oncology Division, Weill Medical College of Cornell University, 413 E. 69th St., New York, NY 10021; 4 Biochemistry Doctoral Program, The Graduate School, City University of New York, New York, NY, 10468; 5 Department of Biological Sciences, City University of New York, Staten Island, 2800 Victory Boulevard, Staten Island, NY 10314; 6 Microenvironment and Metastasis Laboratory, Department of Molecular Oncology, Spanish National Cancer Research Centre (CNIO), Madrid, E28029, Spain

## Abstract

Microvesicles (MVs) are lipid bilayer-covered cell fragments that range in diameter from 30 nm–1uM and are released from all cell types. An increasing number of studies reveal that MVs contain microRNA, mRNA and protein that can be detected in the extracellular space. In this study, we characterized induced pluripotent stem cell (iPSC) MV genesis, content and fusion to retinal progenitor cells (RPCs) *in vitro*. Nanoparticle tracking revealed that iPSCs released approximately 2200 MVs cell/hour in the first 12 hrs with an average diameter of 122 nm. Electron and light microscopic analysis of iPSCs showed MV release via lipid bilayer budding. The mRNA content of iPSC MVs was characterized and revealed the presence of the transcription factors Oct-3/4, Nanog, Klf4, and C-Myc. The protein content of iPSCs MVs, detected by immunogold electron microscopy, revealed the presence of the Oct-3/4 and Nanog. Isolated iPSC MVs were shown to fuse with RPCs *in vitro* at multiple points along the plasma membrane. These findings demonstrate that the mRNA and protein cargo in iPSC MVs have established roles in maintenance of pluripotency. Building on this work, iPSC derived MVs may be shown to be involved in maintaining cellular pluripotency and may have application in regenerative strategies for neural tissue.

The release and fusion activity of microvesicles (MVs) have been analyzed from a number of cell types, including tumor cells, embryonic stem cells, neural and epithelial cells^1^,^2^,^3^,^4^,^5^ and have been isolated from bodily fluids including cerebral spinal fluid, blood and urine^6^,^7^. MVs can be categorized based on size, site of origin and mechanism of formation as either microparticles (100 nm–1 um) or exosomes (30–100 nm)^8^,^9^,^10^. While microparticles bud directly from the plasma membrane, exosomes are formed by inward budding of endosomal membranes, intracellular multivesicular body formation, plasma membrane fusion and exocytotic release^10^,^11^,^12^,^13^. Following release, MVs can circulate locally in the extracellular space adjacent to the cell of origin, or move some distance by diffusion in biological fluids^9^,^14^. Both microparticles and exosomes released into the extracellular environment generate a heterogeneous population containing a range of genetic information^15^,^16^. Although the significance of microvesicle genesis and transfer remains largely undefined, a growing number of studies predict that MV signaling represents a novel mechanism of bi-directional genetic transfer and communication^17^,^18^,^19^.

Microvesicles have unique molecular compositions derived from the type and activation state of the cell of origin and have been shown to contain cell specific subsets of proteins, mRNA, microRNA and organelles^20^,^21^. Embryonic stem cell (ES) derived MVs have been shown to contain ES specific mRNA with the potential to influence target hematopoetic progenitor cell gene expression. Hematopoietic progenitor cells co-cultured with ES derived MVs exhibited enhanced survival and upregulated expression of pluripotent genes^22^,^23^. Also, MVs from adult human bone marrow and mesenchymal stem cells were shown to horizontally transfer mRNAs to recipient kidney tubular cells, contributing resistance to apoptosis and repair of acute kidney injury^24^. Similarly, MVs from human liver stem cells were shown to accelerate the morphological and functional recovery of liver tissue in a rat hepatectomy model^25^.

In this study, mouse iPSC derived MVs were characterized for cell surface release, morphology and molecular composition. We demonstrate that iPSC derived MVs contain a group of iPSC-specific pluripotent transcription factors. We also evaluated iPSC-derived MVs release rate and fusion to target cells *in vitro*. Results from this study suggest that iPSC derived MVs are vehicles for transfer of pluripotent genetic material to target cells with potential application toward regenerative strategies in neural tissue. Analysis of iPSC-derived MVs may enhance understanding of disease pathogenesis and be useful in developing personalized medicine.

## Materials and Methods

### Cell culture

All cells were cultured under sterile conditions and maintained in a 95% O_2_/5% CO_2_ humidified incubator at 37 °C. Primary mouse Nanog-GFP iPSCs were purchased from Stemgent (Cambridge, MA, USA) and cultured in Knockout DMEM (GIBCO, Invitrogen), supplemented with 15% fetal bovine serum (FBS; Sigma), 2 mM L-glutamine, 0.1 mM nonessential amino acids, 0.1 mM β-mercaptoethanol, 5 mg/ml penicillin/100 mg/ml streptomycin, and leukemia inhibitory factor (1 × 10^7^ U/ml). The iPSCs used for MV analysis were plated and cultured without MEFs on 0.1% gelatin-coated T75 culture flasks. Actin promoter-GFP mouse RPCs were isolated from post-natal day one mice and cultured as previously described^26^.

### Cell membrane labeling

To visualize lipid membranes, iPSCs were labeled with the TRITC fluorescent lipophilic dye PKH26 (Sigma). iPSCs (2 × 10^7^ cells) were suspended in PKH26 dilutent-C and mixed with 4 × 10^−6^ M PKH26 dye and incubated at 25 °C for 5 min. The staining reaction was quenched by addition of an equal volume of DMEM supplemented with 1% BSA. Labeled iPSCs were then centrifuged, and re-suspended in pre-warmed media for further analysis.

### Isolation of microvesicles

Microvesicles were isolated using a modified protocol based on previous work by Yuan *et al.*^8^. Exosomes in FBS were depleted by first centrifuging FBS at 110,000 × *g* for 70 min and then filtering using a 0.2 μm pore size filter. To collect iPSC MVs, cells were cultured from 24–48 h, and conditioned media was collected and transferred to centrifuge tubes (polypropylene conical bottom) and centrifuged at 500 × *g* for 10 min to pellet cells at room temperature; supernatant was collected and centrifuged at 10,000 × *g* for 20 min at 4 °C (Beckman Ultracentrifuge, Rotor 60ti) to remove cell fragments and debris; final supernatant was spun at 100, 000 × *g* for 70 min at 4 °C to pellet the microvesicles. Microvesicles were resuspended in phosphate-buffered saline (PBS) and stored at −80 °C for further analysis.

### NanoSight analysis of microvesicle size and concentration

Microvesicle size and number were assessed with NanoSight NS500 system. From 12–48 h after culture in T-75 flasks, conditional media was collected and transferred to centrifuge tubes. Collection of supernatant was identical to previously described isolation of microvesicles, except supernatant before 100, 000 × *g* ultracentrifugation was used for NanoSight analysis. Control media, non-conditioned, was processed at the same time. Final supernatant was diluted at 1:20 in PBS and 1ml was used for NanoSight analysis. The NanoSight system uses a laser light source to illuminate nano-scale particles, detected individually as light-scattered points moving via Brownian motion. Polydispersity was quantified, and we used Nanoparticle Tracking Analysis (NTA) software 2.3 to track and size nanoparticles on an individual basis. Results are displayed as a frequency sized distribution graph describing the number of particles per ml. The concentration of released MVs was calculated to determine average number of MVs with standard deviation in conditioned medium at 12 h, 24 h and 48 h reported and compared using a Student’s t-test.

### Preparation of PKH26-labelled iPSC samples for confocal microscopy

After labeling iPSCs with fluorescent PKH26, cells were incubated at 37 °C for 3 h on glass cover slips. iPSCs were then fixed on coverslips with 4% PFA for 5 min and washed with PBS 3 times at 10 min intervals. The samples were imaged at 40xusing a Leica SP2 AOBS confocal microscope. Excitation was achieved using a HeNe laser. The excitation wavelength was 543 nm and the emission range was 553–650 nm. Z-stacks were obtained and a 3-dimensional reconstruction was done using Nikon Elements software.

### Transmission electron microscopy (TEM)

Cultured iPSCs (4 × 10^7^ cells) were fixed in 2.5% glutaraldehyde with 4% paraformaldehyde for 2.5 h and washed in PBS for 2–4 h. Cells were post-fixed in osmium tetraoxide for 30 min, and washed with distilled water and subsequently dehydrated using increasing ethanol concentrations (70%, 85%, 95% and 100%), for 10 min each, followed by immersion in propylene for 20 min, two times. Next, cells were infiltrated with a 1:1 mixture of propylene oxide and Spurr’s Resin for 1 hand then left in 100% Spurr’s Resin overnight. They were then embedded in Beem Capsules using fresh Spurr’s Resin and left in an oven at 70 °C to polymerize. Excess resin was trimmed and 90 nm sections of cells were made on a Leica Ultracut Ultra microtome. Sections were placed on 200 mesh copper grids, stained with saturated uranylacetate in 50% ethanol for 6 min, followed by rinsing in water and staining for 90 s in lead citrate. Grids were then washed in water, dried on filter paper and viewed under a FeiTecnai transmission electron microscope rated at 80 kV. Images were obtained using an AMT camera with AMT digital software.

### Preparation of microvesicles for TEM

Microvesicles released from iPSCs were isolated from 50 ml of culture supernatant using differential centrifugation. Then 5 μl of suspension containing isolated MVs was dropped on a Zoo-mesh Carbon Formuar grid; MVs were allowed to absorb for 20 min at room temperature. Excess suspension was wicked off and grids were submerged in 25% gluturadeliyde/4% PFA with 25% tannic acid in PBS for 10 min. Grids were then washed in distilled water and viewed on a FeiTecnai transmission electron microscope, operated at 60kv. Digital images were obtained using an AMT digital camera and software.

### Preparation of microvesicles for scanning electron microscopy (SEM)

Microvesicles released from iPSCs were isolated from 50 ml of the culture supernatant using differential centrifugation. The isolated MVs sample was added to membrane filter discs and incubated in 3% glutaraldehyde (dissolved in PO_4_ buffer) for 1 h at room temperature. Glutaraldehyde was then aspirated, and the MVs sample was washed with PO_4_ buffer 4 times, at 10 min. intervals. To dry the sample, PO_4_ buffer was exchanged with ethanol. To avoid osmotic shock, ethanol concentration was gradually increased (10, 30, 50, 70, and 90%). The final step was performed in 100% ethanol for 1 h with three changes of ethanol. Samples were dried with liquid CO_2_ and sputtered with gold. Samples were imaged using a ZeissSupra55VP scanning electron microscope.

### Total RNA purification

Total RNA from iPSCs and microvesicles was isolated using a Max-96 total RNA kit (Life Technologies). Briefly, 20 μL of bead mix was added to iPSCs or MV pellets, and shaken for 5 min. Then, the RNA was magnetically captured by the RNA binding beads and washed with 150 μL wash solutions 1 and 2, followed by shaking for 1 min per wash. 50 μL of Diluted TURBO DNase was added to the sample followed with shaking for 10–15 min at room temperature. After adding 100 μL of RNA rebinding solution, the sample was shaken for 3 min. 150 μL of wash solution 2 was added twice to the sample, followed by shaking for 2 min. Next, 50 μL of elution buffer was added to the sample followed by vigorous shaking for 3 min. The supernatant was transferred to a nuclease-free container. Isolated RNA was measured for quality using a Nano drop ND/1000 spectrophotometer and analyzed by 2% gel electrophoresis.

### Real-time PCR analysis

Total RNA (500 ng) from MVs and iPSCs was reverse-transcribed to generate cDNA using AMV first strand cDNA synthesis kit (New England Biolabs). 10 ng of reverse-transcribed single strand cDNA was used as a template for real-time PCR in 50 μL of RT-PCR mix. Forty cycles of PCR were performed on cDNA samples using SYBR Green ER qPCR ER Supermix (Invitrogen) and six primers including: c-Myc, Klf4, Nanog, β-actin, GAPDH and Oct-3/4. The PCR reaction consisted of an initial enzyme activation step, at 95 °C for 10 min, followed by 40 cycles at 95 °C for 15 s and at 60 °C for 60 s, then followed by melting curve analysis. RT-PCR was carried out in triplicates for each pair of primer. Student’s t-test was used to identify significant differences in mRNA, Ct values, between iPSCs and MVs and p values of less than 0.05 were considered statistically significant.

### Immunoelectron microscopy

Five μl of resuspended 2% paraformaldehyde fixed MVs were put on glow discharged formvar-carbon coated nickel grids. After washing with PBS, the grids were incubated with 50 mM glycine/PBS for 3 min. The grids were blocked for 10 min with either 1% coldwater fish skin gelatin (Sigma-Aldrich) for the surface immunolabeling (Tsg101, Abcam), or 5% BSA, 5% goat serum, 0.1% cold-water fish skin gelatin and 0.1% saponin in PBS for inner membrane protein labeling (Nanog and Oct4, Abcam). Primary antibodies in blocking solution for Tsg101 or in antibody incubation buffer (0.1% BSA and 0.1% saponin) for Nanog and Oct4 were applied for 2 hours at room temperature. Controls were prepared in the absence of primary antibodies. After washing with PBS, Nanogold-labeled Fab’ anti-rabbit or anti-mouse (Nanoprobes, NY), or 5 nm, 10 nm gold conjugated goat anti-mouse antibodies (Ted Pella Inc. Redding, CA) were applied in the correlated antibody incubation buffer for 1 hour. The grids were then washing with PBS, fixed in 1% glutaraldehyde for 5 min. After thoroughly washed with distilled water, the grids were either directly go to methylcellulose embedding for 5 nm or 10 nm gold, or continue with silver enhancement for nanogold. For the silver enhancement, the grids were washed with 0.02 M sodium citrate (pH 7.0), and performed silver enhancement in the dark using HQ Silver enhancement kit (Nanoprobes, NY) at room temperature for 8 min. After washing with distilled water, the grids were contrasted and embedded in a mixture of 3% uranyl acetate and 2% methylcellulose in a ratio of 1–9. Stained grids were examined under Philips CM-12 electron microscope and photographed with a Gatan (1k×1k) digital camera. All antibodys were purchased from Abcam, USA. Anti-Tsg101 (1:200), Anti- Nanog (1:400) and Anti-Oct4 (1:200) were diluted according to manufacturer’s instruction.

### Transfer and fusion of PKH26-labelled iPSC microvesicles to RPCs

Microvesicles from PKH26-labelled iPSCs, were isolated from 50 ml of culture supernatant using differential centrifugation. PKH26-labelled microvesicles were incubated with retinal progenitor cells (RPCs) in culture at 37 °C for 3 h. RPCs were used to demonstrate iPSC-MV binding within a robust neural progenitor population. RPCs with fused PKH26-labelled microvesicles were fixed in 4% PFA for 20 min and washed with PBS 3 times, 10 min each. Samples were imaged using a Leica SP2 AOBS confocal microscope with 40× oil immersion objective and digital zoom. A HeNe laser was used for PKH26-labelled MVs. The excitation wavelength was 543 nm and the emission range was 553–650 nm. A 488 Argon Laser was used to image for GFP RPCs.

## Results

### Size and Concentration of microvesicles derived from iPSCs

**Figure 1:**
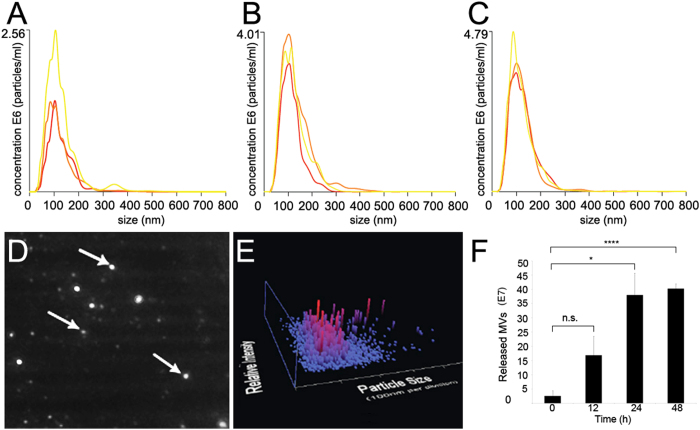
iPSC microvesicle diameter and release rate. (**A–C**) MV nanometer diameter range and concentration were derived from iPSC conditioned media at 12 h, 24 h and 48 h, respectively. MV diameters and concentrations for three samples at each time point are indicated in colored traces. Mean diameters and standard deviation for each time point remained consistent; 12 h 122 ± 2.3 nm, 24 h 124 ±6.0 nm, 48 h 122 ± 2.2 nm. (**D**) Arrows show light-scattering of individual MVs from a single frame of Nanosight tracking analysis at 24 h. (**E**) 3D plot showing MV size/relative intensity. (**F**) Average number of MVs in conditioned media at 12 h = (1.69 ± 0.66)* 10^8^, 24 h = (3.80 ± 0.77)* 10^8^ and 48 h = (4.20 ± 0.40)* 10^8^. Error bars represent standard deviation. Student’s t-test was performed to analyze iPSC release rates. p < 0.05 was considered statistically significant. “ns” represents not significantly different.

Microvesicles isolated from iPSCs were analyzed using nanoparticle tracking analysis technology (NTA). NTA tracking allowed a robust analysis of secreted vesicle size and release rate (Fig. 1). Data shows chat iPSC released MVs have diameters that fall within the range reported for secreted microparticles (100 nm–1 um)^8^,^10^. MVs derived from iPSC conditioned media at three time points, 12, 24 and 48 h showed stable mean diameters of 122 ± 2.3 nm, 124 ± 6.0 nm and 122 ± 2.2 nm, respectively. The concentration of released MVs was calculated using a Student’s t-test and the average number of MVs in conditioned medium at 12 h = (1.69 ± 0.66)* 10^8^, 24 h = (3.80 ± 0.77)* 10^8^ and 48 h = (4.20 ± 0.40)* 10^8^ (Fig. 1F). The number of MVs released per cell/hr was also calculated after data was normalized to control media. At 12 h post-plating of 1 × 10^6^cells, individual iPSCs had released an estimated 2200 ± 884 MVs; at 24 h the number increased to 5000 ± 1023 MVs and at 48 h 5300 ± 220 MVs were released (data not shown). A sample of a 20 s Nanosight video, from which the 12 h time point MV brownian movement and tracking data were derived, is provided in supplemental movie 1 (S1).

**Figure 2:**
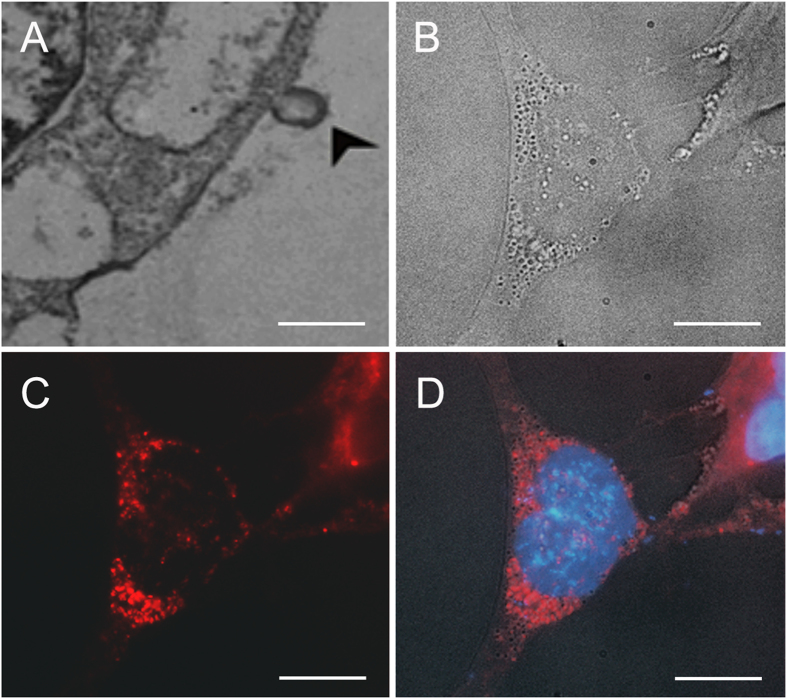
Analysis of cell surface microvesicle formation. (**A**) TEM image of exosome formation at the iPSC surface (Fig. 2A, arrowhead), scale: 500 nm. (**B**) Bright field image of iPSC with surface topology suggestive of vesicle formation at the cell membrane, (**C**) Following PKH26 (red) lipophilic labeling, emerging vesicles at the iPSC lipid bilayer appear concentrated near the soma with observable variations in size, (**d**) DAPI nuclear labeling with overlay of (**B,C**), scale: 10 μm.

TEM analysis revealed release of MVs from iPSCs (Fig. 2A). TEM shows a microparticle (>100 nm) budding from the plasma membrane into the extracellular space (Fig. 2A, arrowhead). To further confirm lipid involvement in release of MVs from iPSCs, we utilized confocal analysis of PKH26 labeled cell membrane and imaged the release of MVs emerging from iPSCs (Fig. 2B–D). Our analysis suggests that both exosomes and microparticles were released from PKH26-lablelled iPSCs. Exosome budding appeared across the plasma membrane with release occurring periodically in cluster sites.

### Morphology of microvesicles isolated from iPSCs

**Figure 3:**
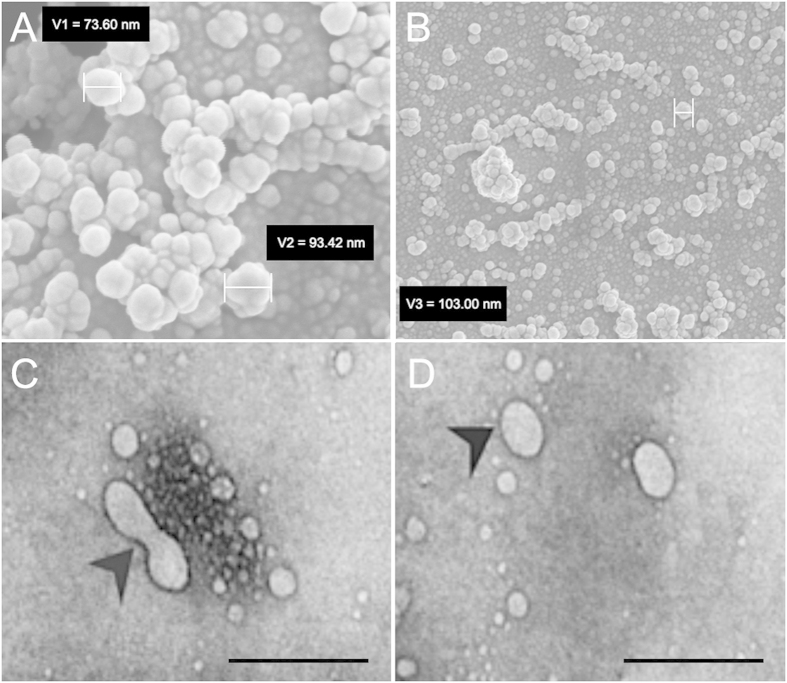
SEM and TEM analysis of iPSC derived microvesicles. SEM analysis of MV populations isolated from iPSCs revealed a heterogenous population of spheroid vesicles ranging in size from 30–300 nm. (**A**) SEM of an MV population with a sample microparticle measured with a diameter of 103 nM, scale bar: 200 nm. (**B**) A higher magnification SEM image showing measured exosomes with diameters of 73.60 and 93.42 nm. (**C**) TEM analysis of MVs isolated from iPSC supernatant showed characteristic cup-shaped morphology and size (arrowhead) and (**D**) spheroid exosome (arrowhead) scale bar: 100 nm.

The morphology and size of isolated iPSC microvesicles were analyzed using SEM and TEM. Visualization of iPSC MVs isolated via ultra centrufugation and imaged using SEM revealed heterogeneous spheroid morphologies with sizes ranging from 30 to 300 nm. SEM shows microparticles (Fig. 3A) to be approximately 100–300 nM, while exosomes ranged from 30–95 nm (Fig. 3B). The smooth shape of the MV surface, observed on the SEM image, can be attributed to the physical properties of the phospholipid bilayer. Using TEM analysis Fig. 3D shows a sample of an isolated microparticle with a diameters of approximately 130 nM. A second image shows an exosome with the characteristic spheroid shape and a diameter of approximately 70 nM (Fig. 3C). TEM images revealed that iPSC exosomes have a cup-shaped appearance, which uniquely differentiates them from microparticles. Microparticles appeared consistently spheroid in shape. This level of TEM analysis aligns with previous studies describing microparticles as heterogeneous in shape and larger than exosomes with diameters reaching up to 1 μm^27^,^28^.

### RNA and protein identified in microvesicles derived from iPSCs

**Figure 4:**
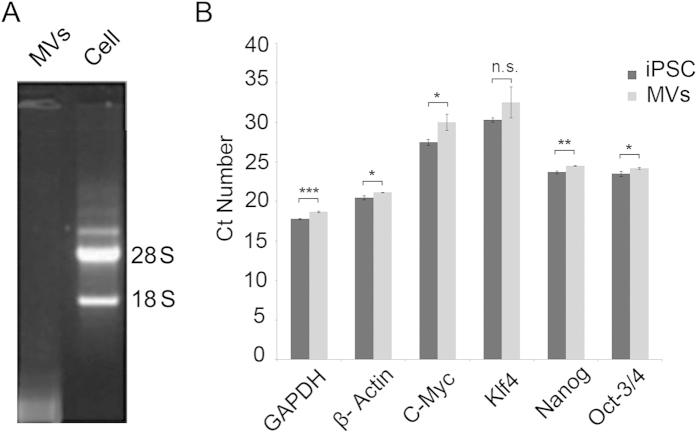
iPSC microvesicles contain pluripotent transcription factor mRNA. (**A**) Total RNA isolated from iPSC derived MVs lacks 28 S and 18 S rRNA as revealed through 2% denaturing agarose gel loaded with total RNA from iPSC MVs and iPSCs. (**B**) To verify the presence of selected iPSC derived mRNA, MV mRNA was analyzed using real-time PCR and contained factors involved in the maintenance of iPSC pluripotency including Oct-3/4, Nanog, Klf4, and C-Myc, Positive controls β- actin and GAPDH were present in iPSCs and MVs. Significant differences between cells and MVs are denoted with asterisks: *(p < 0.05), **(p < 0.01), ***(p < 0.001), ****(p < 0.0001); “ns” - not significantly different.

The total RNA profile of iPSC MVs was determined by native agarose gel electrophoresis. As displayed in Fig. 4A, the 18 S and 28 S ribosomal RNA bands are clearly visible in the total RNA profile of iPSCs (lane2); in contrast, the total RNA from iPSC derived MVs does not contain 28 S and 18 S ribosomal RNA (lane1). The largest fraction of total RNA isolated from the MV population appeared as a lower molecular weight band suggesting the presence of mRNA and small RNA species. We then confirmed the presence of selected mRNA species in iPSC MVs. Isolated total RNA from iPSC MVs (1 μg) was reverse-transcribed and used as a template for amplification using the primers shown in Table 1. The house keeping genes β-actin and GAPDH were tested as positive controls and were present in both iPSCs and MVs (Fig. 4B). Both β-actin and GAPDH displayed significantly lower expression level in MVs than in iPSCs (p < 0.05). Due to baseline differences in expression levels, β-actin and GAPDH were not normalized to in our experiments. To compare expression levels of transcription factors between iPSCs and MVs, the same amounts of RNA were used in reverse-transcription. Four transcription factors including Oct-3/4, Nanog, Klf4, and C-Myc were found in both cells and isolated MVs, but expression levels were lower in MVs than in iPSCs (Fig. 4B). With the exception of Klf, each RNA species analyzed was present in higher levels in cells than MVs. The rRNA content was also compared between MVs and iPSCs of origin, showing MVs lack 18 S and 28 S rRNA species (Fig. 4A).

**Figure 5:**
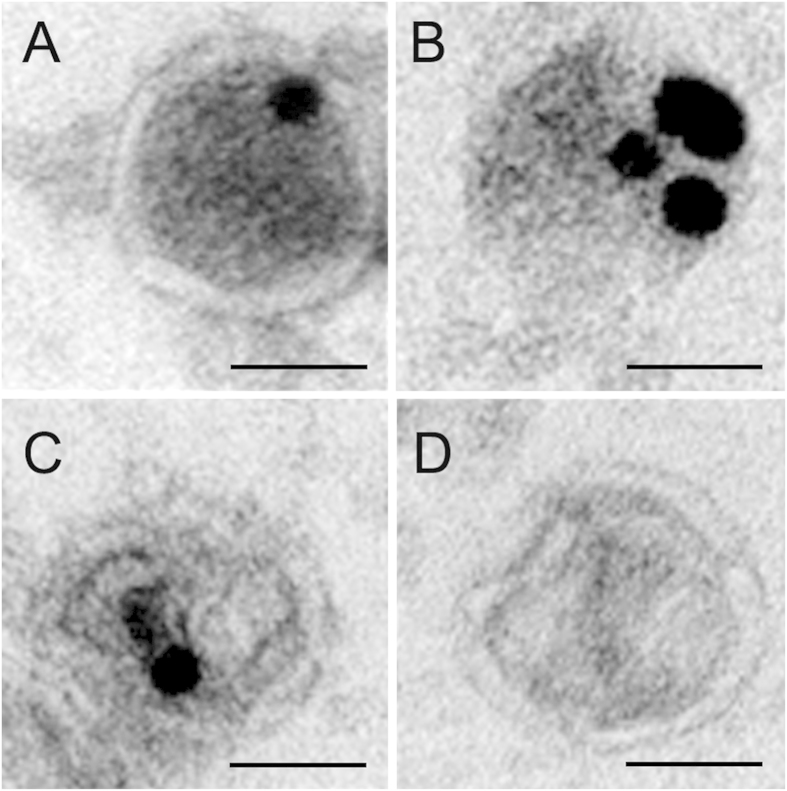
Immunogold EM analysis of Microvesicle Protein Content. Immunogold EM images reveal the presence of (**A**) the homeodomain transcription factor Nanog, (**B**) the homeodomain transcription factor Oct-3/4, (**C**) the tetraspanprotein Tsg101, a canonical MV marker, and (**D**) control. Each pluripotent protein identified within MVs is involved in undifferentiated iPSC self-renewal. Samples were viewed under a FeiTecnai Transmission Electron Microscope. Scale: 50 nm.

The protein content of iPSC MVs was visualized using immunogold TEM. In Fig. 5, black punctate regions indicate immunogold labeling of the homeodomain transcription factor Nanog, the homeodomain transcription factor Oct-3/4 and the the tetraspanprotein TSG101 protein within MVs. Each pluripotent protein identified within MVs has established roles in maintaining undifferentiated iPSC self-renewal.

### Fusion of microvesicles derived from iPSCs with RPCs *in vitro*

**Figure 6:**
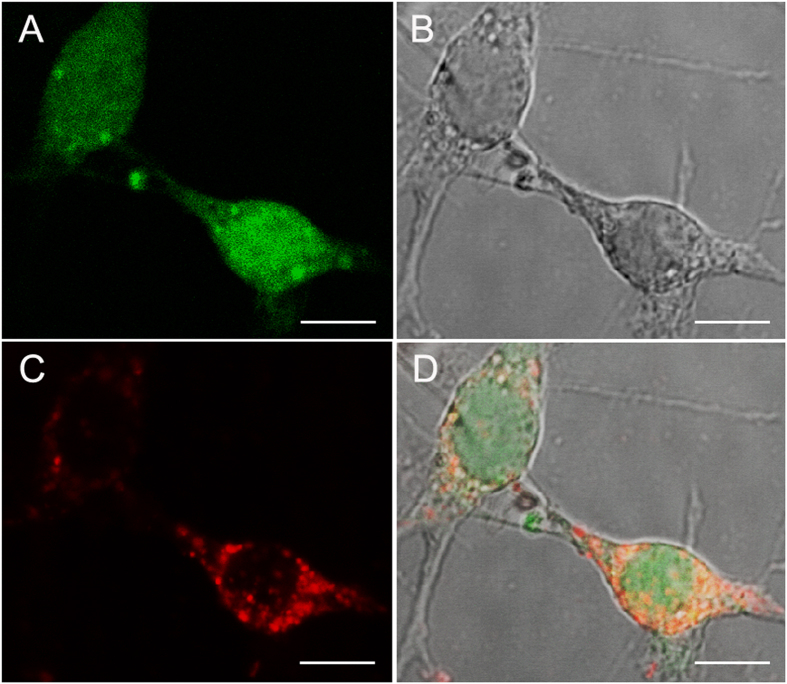
iPSC microvesicles fuse to target cells *in vitro*. (**A**) iPSC MV co-cultured GFP^+^ RPCs remain viable expressing GFP ubiquitously throughout the cytoplasm and robust branching morphology. (**B**) Dendritic processes and cell-to cell contacts visible using phase contrast. (**C**) MVs isolated from iPSCs labeled with the red fluorescent lipophilic dye PKH26fuse to GFP+RPCs. (**D**) Overlay (**A**–**C**). scale 10 μm.

The red fluorescent lipophilic membrane dye PKH26 was used to label isolated iPSC derived MVs, which were then incubated with target RPCs to evaluate MV docking and fusion (Fig. 6). Following three hours of co-incubation, IPSC derived MVs attached to RPCs and a percentage remained adherent flowing multiple rinses with warmed media. Imaging revealed the binding of single and clustered MVs across the surface of GFP RPCs with varying morphologies, as single cells and within larger colonies. MV binding appeared to be facilitated via lipid fusion as MVs were visible only on cells and not on cell-free culture surfaces following rinsing. A sample z-stack 3D confocal reconstruction of MV fusion to GFP RPCs is available in supplemental movie 2 (S2).

## Discussion

In this study we observed that iPSC MV generation and release are consistent with previous studies describing components of microvesicle biogenesis^14^,^29^. We discovered that the cargo of iPSC derived MVs incudes mRNA and protein species involved in self-renewal and pluripotentcy^30^,^31^,^32^. In addition, SEM and TEM analysis of iPSC derived MVs was consistent with previous findings, describing microparticles as heterogeneous in shape, and exosomes with cup-shaped morphologies^22^,^33^.

In the process of electron microscopic analysis, we observed nano sized, grain-like structures (>20 nm) in the background of SEM images (Fig. 3A). These structures are gold grains formed as a result of gold sputtering during sample preparation for SEM. Additional studies may advance MV analysis by use of flow cytometry. An advantage of flow cytometry is that no sputtering procedures are needed. A disadvantage would be that the structural features of the MVs would remain indistinct^34^ and most systems cannot reliably acquire data on the smaller size MVs (>500 nm). To address these current technical challenges in the field of MV analysis, the Scientific Standardization Committee of the International Society on Thrombosis and Haemostasis are evaluating standardized protocols for both MV isolation and analysis^9^.

Our findings support existing data describing the process of microparticle and exosome genesis. However, the signaling mechanisms of initiation and termination of MV genesis remains to be fully elucidated^10^. Using TEM, we showed how microparticles were formed by the process of lipid bilayer budding^14^. We also observed exosome secretion from multivesicular bodies (data not shown). Among membranous vesicles, exosomes are the only type that originate from intracellular compartments, such as the MVBs^33^. According to a previous study, MVBs either fuse with lysosomes to degrade their bioactive load or fuse with the plasma membrane to release their exosomes into the extracellular environment^11^.

In agreement with a number of studies, our findings support that MVs contain a subset of mRNA and protein derived from the cell type of origin^3^,^8^,^20^. In particular, we have shown that iPSC MVs contain mRNA and protein, which encode for and function as transcription factors critical to maintaining iPSC pluripotency. In addition, iPSC MVs are enriched in the transcription factors Nanog and Oct-3/4, important for iPSC self-renewal. Current understanding of cell targeting of subsets of RNA and proteins to MVs remains limited^35^. A recent study suggest that ES MVs transfer a specific subset of miRNAs and mRNA in a highly selective manner possibly regulated by proteins found MV mebranes^8^. Cargo selection for MVs may also be understood by comparing the expression profile of mRNA and miRNA in the cells of origin and the presence of ribonucleo proteins that function in the intracellular transport^13^. For example, MVs released from MSCs have been shown to contain both cell specific mRNA and ribonucleo proteins that function in RNA storage, transport and stability. Current studies suggest that MV cargo uptake is facilitated by clathrin-mediated processes with ribonucleo proteins targeting miRNA and mRNA and N-linked glycosylation directing protein cargo^13^,^36^,^37^.

In this work, our confocal analysis confirms earlier studies showing that an essential mechanism through which MVs communicate with target cells is by fusing directly with the plasma membrane (Fig. 6). Here FITC labeled MVs remained bound to, and potentially became internalized in, RPCs following a brief incubation. Our 3D confocal reconstruction of MV fusion inS2 shows robust fusion at several points on target RPCs. MVs have been reported to release their contents into the cytoplasm of target cells with the potential of influencing gene expression states, viability and plasticity^15^,^19^. Recent findings indicate that fusion is the initial step required for horizontal transfer of MV genetic cargo as well as proteins, phospholipids and organelles^38^,^39^.

Further analysis of the process of iPSC MV transfer of pluripotent transcripts and protein will build on data presented here. MVs derived from ES and iPSCs may influence expression patterns toward pluripotentcy in target cells^2^. Recently it has been shown that ES MVs can alter the gene expression of retinal Muller cells contributing to trans differentiation, an initial step toward regeneration^40^. The finding that ES MVs may switch on early programs of pluripotency suggests that MVs may serve as therapeutic agents to restore the regenerative potential of the retina. In line with this research, iPSCs may be developed as a patient specific vehicle for delivery of genetic cargo toward nervous system repair. In support of the *in vivo* neuro protective potential of MVs in retinal disease, a recent study has examined the role of exosome transfer of crystallin (a biomarker in age related macular degeneration) between retinal pigment epithelial cells and photoreceptors. The authors demonstrated that MV-secreted crystalline was neuro protective for photoreceptors^41^. Additional studies suggest that miRNAs may be potential therapeutic targets for age related macular degeneration^42^. Since it may be harmful to inject naked siRNAs into the eye, MVs may also be useful to transfer miRNA to treat retinal disease^8^.

## Additional Information

**How to cite this article**: Zhou, J. *et al.* Characterization of Induced Pluripotent Stem Cell Microvesicle Genesis, Morphology and Pluripotent Content. *Sci. Rep.* **6**, 19743; doi: 10.1038/srep19743 (2016).

## Supplementary Material

Supplementary Movie S1

Supplementary Movie S2

## Figures and Tables

**Table 1:** Primers used for real-time PCR.

Primer pairs	Sequence (5′–3′)	Amplicon (bp)
C-Myc	CAGAGGAGGAACGAGCTGAAGCGC	228 bp
	TTATGCACCAGAGTTTCGAAGCTGTTCG	
Nanog	AGGGTCTGCTACTGAGA TGCTCTG	228 bp
	CAACCACTGGTTTTTCTGCCACCG	
Oct-3/4	CTGAGGGCCAGGCAGGAGCACGAG	485 bp
	CTGTAGGGAGGGCTTCGGGCACTT	
Klf4	CACCATGGACCCGGGCGTGGCTGCCAGAAA	739 bp
	TTAGGCTGTTCTTTTCCGGGGCCACGA	
β- actin	TGTTACCAACTGGGACGACA	150 bp
	ACCTGGGTCATCTTTTCACG	
GAPDH	TGGCAAAGTGGAGATTGTTGCC	150 bp
	AAGATGGTGATGGGCTTCCCG	
